# Finerenone in diabetic kidney disease: a new frontier for slowing disease progression

**DOI:** 10.3389/fmed.2025.1580645

**Published:** 2025-06-02

**Authors:** Ibrahim S. Alhomoud, Mohamed A. Albekery, Razan Alqadi, Amal Alqumia, Riaz A. Khan, Muthana Al Sahlawi, Mohammed Yousef Al Mulhim

**Affiliations:** ^1^Department of Pharmacy Practice, College of Pharmacy, Qassim University, Qassim, Saudi Arabia; ^2^Department of Pharmacy Practice, College of Clinical Pharmacy, King Faisal University, Al-Ahsa, Saudi Arabia; ^3^Department of Pharmacy, King Saud Hospital, Unaizah, Saudi Arabia; ^4^Department of Pharmacy, King Fahd Specialist Hospital, Buraydah, Saudi Arabia; ^5^Department of Medicinal Chemistry and Pharmacognosy, College of Pharmacy, Qassim University, Buraydah, Saudi Arabia; ^6^Department of Internal Medicine, College of Medicine, King Faisal University, Al Hofuf, Saudi Arabia

**Keywords:** finerenone, diabetes mellitus, diabetic kidney disease (DKD), chronic kidney disease (CKD), mineralocorticoid receptor antagonists (MRA)

## Abstract

Diabetic kidney disease (DKD) represents a substantial health burden for patients with type 2 diabetes mellitus (T2DM), markedly increasing morbidity and mortality. The cornerstone of DKD treatment remains renin-angiotensin system (RAS) blockade and risk factor control, such as blood pressure management, glycemic control, albuminuria reduction, and lipid management. However, treatment strategies have expanded to include sodium-glucose cotransporter-2 (SGLT-2) inhibitors and glucagon-like peptide-1 (GLP-1) receptor agonists, which have proven effective in slowing kidney disease progression when combined with RAS inhibitors. Finerenone, a non-steroidal mineralocorticoid receptor antagonist (MRA), represents a novel approach to the management of DKD. It offers unique pharmacokinetic and pharmacodynamic properties compared with steroidal MRAs such as spironolactone and eplerenone. This review addresses the evolving landscape of diabetic kidney disease management, with a focus on finerenone’s distinct pharmacologic properties, structural characteristics, and clinical implications.

## 1 Introduction

Chronic kidney disease (CKD) is defined as persistent abnormalities in kidney structure or function, indicated by an estimated glomerular filtration rate (eGFR) of ≤ 60 mL/min/1.73 m^2^ for a minimum of 3 months (1). As of 2023, the Centers for Disease Control and Prevention (CDC) estimates that approximately 14% of United States adults-equivalent to 35.5 million individuals-are affected by CKD (2). Approximately 50% of people with type 2 diabetes mellitus (T2DM) and 33% of those with type 1 diabetes (T1DM) experience kidney disease as a result of their condition and/or other associated illnesses, including hypertension (HTN), dyslipidemia, obesity, intra-renal vascular disease, glomerular atherosclerosis, kidney ischemia, or age-related nephron loss (3). Diabetic kidney disease (DKD) is characterized by abnormal urinary albumin excretion, with an albumin-to-creatinine ratio (ACR) of 30–299 mg/g for moderate albuminuria and ≥300 mg/g for severe albuminuria (4). Table 1 shows the CKD staging system with risk levels ranging from low to very high based on eGFR and albuminuria levels.

**TABLE 1:** Chronic kidney disease (CKD) staging based on eGFR and albuminuria levels (4).

eGFR (G) ml/min per 1.73 m^2^		Albuminuria (A)		
		A1	A2	A3
		Normal to mildly increased	Moderately increased	Severely increased
		<30 mg/g <3 mg/mmol	30–299 mg/g 3–29 mg/mmol	≥300 mg/g ≥30 mg/mmol
G1	≥90			
G2	60–89			
G3a	45–59			
G3b	30–44			
G4	15–29			
G5	<15			

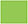
 Low risk; 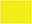 Moderately increased risk; 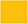 High risk; 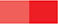 Very high risk.

Diabetic kidney disease substantially increases cardiovascular risk and the economic burden on healthcare systems (5). Patients with diabetes without kidney disease have a 10-year standardized cumulative all-cause mortality rate of 11.5% (95% CI, 7.9 to 15.2%). However, when diabetes is associated with kidney disease—defined as albuminuria (ACR ≥ 30 mg/g) and/or impaired eGFR (≤60 ml/min/1.73 m^2^)—the mortality rate increases significantly to 31.1% (95% CI, 24.7 to 37.5%) (6). This increase highlights the impact of kidney dysfunction on survival. Among markers of kidney injury, albuminuria is a key determinant that promotes oxidative stress and impaired nitric oxide bioavailability, thereby increasing the risk of cardiovascular events and progression of chronic kidney disease in individuals with diabetes and metabolic syndrome. Beyond serving as a marker of kidney impairment, albuminuria reflects systemic inflammation and endothelial dysfunction, further exacerbating cardiovascular risk (7). A population-based prospective study conducted in Denmark with 626 participants without existing heart or kidney failure demonstrated that albuminuria was associated with a 2.3-fold increased risk of major cardiovascular events (hazard ratio [HR], 2.3; 95% CI, 1.33 to 4.05; *P* = 0.003) (8). Notably, the study found albuminuria to be a stronger predictor of first major cardiovascular events than C-reactive protein (CRP) (8).

The American Diabetes Association (ADA) and Kidney Disease: Improving Global Outcomes (KDIGO) guidelines recommend annual screening for CKD starting 5 years or more after diagnosis in individuals with T1DM. For those with T2DM, the guidelines recommend annual CKD screening at the time of diagnosis (4). Additionally, the KDIGO emphasizes the importance of screening for CKD, particularly for high-risk patients, including those with hypertension and cardiovascular disease (9). However, CKD screening remains considerably underutilized. A nationwide study across the United States found that less than 20% of at-risk individuals with diabetes or hypertension received both guideline-recommended tests, with only 21% undergoing ACR testing (10). These findings were consistent with a retrospective cohort study that involved 192,108 adults diagnosed with hypertension or diabetes in the U.S. and revealed that an estimated 82.5% did not undergo ACR testing (11). The CDC reports that an estimated 90% of adults with CKD, and about one in three individuals with severe CKD, are unaware of their diagnosis (2). Early detection of CKD is essential for initiating timely interventions that can slow disease progression and mitigate complications. The REVEAL-CKD study demonstrated that following a CKD diagnosis, the annual rate of kidney function decline slowed considerably, decreasing from 3.20 ml/min/1.73 m^2^ (95% CI, −3.38 to −3.00) to 0.74 ml/min/1.73 m^2^ (95% CI, −0.96 to −0.53) (10). While early detection and slower progression of CKD are crucial, certain clinical scenarios warrant nephrology referral. Referral is recommended for patients with CKD of uncertain origin, rapidly declining kidney function, or an eGFR ≤ 30 ml/min/1.73 m^2^. Additional indications included complex complications, such as anemia, metabolic bone disease, electrolyte imbalances, resistant hypertension, or a marked rise in albuminuria despite optimal blood pressure management (12).

## 2 Current therapies for managing diabetic kidney diseases

Current DKD treatment emphasizes individualized glycemic and blood pressure targets to optimize patient outcomes as part of a comprehensive approach, including routine monitoring of blood glucose, blood pressure, lipids, and albuminuria. Management should incorporate lifestyle modifications such as dietary restrictions, regular physical activity, and smoking cessation to slow disease progression and improve cardiovascular and kidney outcomes. Given the progressive nature of DKD, early disease management prioritizes stringent targets, aligning with KDIGO guidelines that recommend a systolic blood pressure goal of <120 mmHg (13). To achieve this target, both KDIGO and ADA guidelines recommend titrating angiotensin-converting enzyme (ACE) inhibitors or angiotensin II receptor blockers (ARBs) to the maximum tolerated dose (4). The ADA guideline has set a target to reduce albuminuria by at least 30% in individuals with DKD who have levels at or above 300 mg/g (12). Evidence shows that a 30% reduction in ACR over 2 years is associated with a significantly lower risk of progression to end-stage kidney disease (ESKD), with an adjusted hazard ratio of 0.83 (95% CI, 0.74 to 0.94) (14).

Angiotensin-converting enzyme inhibitors were the first class of medications shown to reduce albuminuria in the 1990s (15, 16). By the early 2000s, ARBs demonstrated therapeutic efficacy comparable to that of ACE inhibitors in reducing albuminuria. However, the concurrent use of ACE inhibitors and ARBs is contraindicated due to an increased risk of hyperkalemia and acute kidney injury (4, 12). Although renin-angiotensin system (RAS) blockade with ACE inhibitors or ARBs remains the cornerstone of DKD management, many patients remain suboptimally treated. Inadequate optimization of RAS blockade results in persistent albuminuria and progressive kidney function decline, leading to higher rates of morbidity and mortality. In a cohort study of 141,252 veterans with CKD and albuminuria, discontinuation of RAS blockade was associated with a 74% increased risk of mortality (HR 1.74; 95% CI, 1.70 to 1.78) and a 47% higher risk of ESKD progression (HR 1.47; 95% CI, 1.26 to 1.71) (17). Consistent with these findings, a retrospective cohort study of 3,909 patients with advanced CKD reported that among individuals experiencing an eGFR decline below 30 mL/min/1.73 m^2^, withdrawal of RAS blockade within 6 months was associated with a 39% increased risk of mortality (HR 1.39; 95% CI, 1.20 to 1.60) and a 37% higher risk of major adverse cardiovascular events (MACE) (HR 1.37; 95% CI, 1.20 to 1.56) (18). The STOP-ACEi trial prospectively evaluated the effects of discontinuing versus continuing RAS inhibitors in 403 patients with advanced CKD (eGFR < 30 mL/min/1.73 m^2^) (19). The trial found no significant difference in the rate of eGFR decline between the two groups; however, it did not assess mortality outcomes. Patients who discontinued ACE inhibitors had a higher incidence of kidney failure or the need for kidney replacement therapy (65% vs. 54%; HR 1.52; 95% CI, 1.07 to 2.16) (19). Although ACE inhibitors and ARBs are foundational therapies in CKD management, clinical data suggest that RAS blockade alone does not fully prevent disease progression, likely due to residual RAS activity and alternative mechanisms contributing to kidney function decline (20). Given these limitations, the need to expand pharmacologic strategies beyond RAS blockade was recognized as essential to further slow DKD progression, leading to the development and integration of novel therapeutic approaches.

Sodium-glucose cotransporter-2 (SGLT-2) inhibitors emerged as one of the first major therapeutic advancements beyond RAS blockade (21). Landmark clinical trials that evaluated the role of SGLT-2 inhibitors in CKD required optimization of RAS blockade with either an ACE inhibitor or an ARB, reinforcing their adjunctive rather than substitutive role (22–24). The effectiveness of SGLT-2 inhibitors in reducing kidney outcomes appears to be consistent regardless of baseline kidney function, with an overall hazard ratio of 0.66 (95% CI, 0.61 to 0.71) Across diverse CKD populations, SGLT-2 inhibitors demonstrated a consistent reduction in the risk of kidney events (HR 0.64, 95% CI, 0.59 to 0.70) (25). The magnitude of risk reduction remained robust across different eGFR strata. However, the kidney benefits were more pronounced in patients with normal to mildly increased albuminuria compared to those with moderate or severe albuminuria (25). These data highlight the importance of early intervention to enhance kidney preservation and improve long-term outcomes. Furthermore, SGLT-2 inhibitors demonstrated consistent benefits on key composite kidney outcomes—including worsening kidney function, progression to end-stage kidney disease, and kidney-related death—regardless of atherosclerotic cardiovascular disease (ASCVD) status in patients with T2DM (26). This suggests a broad therapeutic role across varying cardiovascular risk phenotypes. In addition to kidney protection, SGLT-2 inhibitors provide cardiovascular benefits that are independent of glycemic control. These effects further support their integration into treatment strategies for patients with CKD and cardiovascular comorbidities. The CVD-REAL 3 study validated the real-world effectiveness of SGLT-2 inhibitors in a multinational cohort analysis. The study found that SGLT-2 inhibitor use reduced the risk of a 50% decline in eGFR or progression to end-stage kidney disease by 51% compared to other glucose-lowering therapies (HR 0.49; 95% CI, 0.35 to 0.67) (27). Given their consistent benefits in slowing disease progression and reducing cardiovascular risk, SGLT-2 inhibitors have become an integral component of DKD management. However, significant residual risk persists despite optimizing the patients with RAS blockade and SGLT-2 inhibitors (24, 28, 29).

Glucagon-like peptide-1 (GLP-1) receptor agonists represent a novel therapeutic approach in DKD management. These agents complement RAS blockade and SGLT-2 inhibitors by targeting multiple pathophysiologic mechanisms, including proximal tubule sodium-hydrogen exchanger 3 inhibition, enhanced natriuresis, reduced oxidative stress, and modulation of inflammatory, fibrotic, and glomerular hemodynamic pathways (30, 31). Additionally, GLP-1 receptor agonists exert indirect effects, including weight loss, improved glycemic control, and blood pressure reduction, all of which contribute to enhanced kidney outcomes (32). The kidney protective effects of GLP-1 receptor agonists have been increasingly recognized, initially through exploratory findings or secondary outcomes in clinical trials (33–37). However, the FLOW trial was the first to evaluate kidney outcomes as a primary endpoint in patients with type 2 diabetes and chronic kidney disease (38). In this trial, once-weekly semaglutide 1 mg significantly reduced the risk of kidney disease progression, kidney failure, or cardiovascular mortality by 24% compared with placebo. Due to the clear evidence of efficacy, the trial was terminated early, with a hazard ratio of 0.76 (95% CI, 0.66 to 0.88) for the composite kidney outcome. Given the magnitude of benefit, the U.S. Food and Drug Administration (FDA) recently approved semaglutide to mitigate the risk of kidney disease progression and cardiovascular mortality in adults with T2DM and CKD. Incretin-based therapies beyond semaglutide have also demonstrated positive effects on kidney outcomes in patients with T2DM. A *post hoc* analysis of the SURPASS-4 trial showed that tirzepatide significantly reduced the risk of composite kidney outcomes, including a ≥ 40% eGFR decline from baseline, kidney failure, kidney death, or new-onset macroalbuminuria, with a hazard ratio of 0.59 (95% CI, 0.43 to 0.80) (39). To confirm findings from exploratory analyses, the ongoing TREASURE-CKD trial (NCT05536804) is investigating tirzepatide’s role in CKD management in patients with overweight or obesity, with or without diabetes.

## 3 Finerenone drug discovery perspective: receptor binding, adverse effects, and bioactivity perturbations

The mineralocorticoid receptor (MR) is a key driver in the pathogenesis of CKD and cardiovascular complications. Aldosterone, the principal ligand for MR, is synthesized by the adrenal glands and acts on both epithelial tissues and non-epithelial tissues. In the kidney, aldosterone promotes sodium reabsorption and potassium excretion, leading to fluid retention and hypertension. Beyond its hemodynamic effects, pathological MR activation contributes to oxidative stress, inflammation, and tissue fibrosis, accelerating kidney and cardiovascular complications (40). The clinical significance of MR overactivation has driven the development of mineralocorticoid receptor antagonists (MRAs) as therapeutic agents aimed at mitigating aldosterone-mediated effects and reducing cardiovascular and kidney complications (41). The origins of MRAs were primarily centered on the idea of ‘aldosterone antagonists’. In 1942, studies revealed that deoxycorticosterone, a precursor to aldosterone, induced nephrosclerosis and cardiac hypertrophy in animals (42). This led to the development of the first aldosterone antagonist, spironolactone, which was found to protect rats from aldosterone-induced cardiac necrosis (41).

The first-generation steroidal MRAs such as spironolactone and eplerenone have demonstrated efficacy in reducing proteinuria and slowing disease progression. However, their use is often limited by the risk of hyperkalemia, which is a significant concern in CKD due to impaired potassium excretion. The benefits and risks of steroidal MRAs have been substantiated by a meta-analysis of 31 randomized controlled trials involving 2,767 participants. This analysis demonstrated that MRAs, whether used alone or in combination with ACE inhibitors or ARBs, significantly reduced albuminuria by 24.55% (95% CI, −29.57% to −19.53%) and proteinuria by 53.93% (95% CI, −79% to −28.86%) (43). However, these benefits were accompanied by a 2.6-fold increased risk of hyperkalemia, with a relative risk of 2.63 (95% CI, 1.69 to 4.08) and an average potassium increase of 0.22 mEq/L (43). This safety concern in patients with CKD has driven the development of finerenone, a non-steroidal MRA designed to maintain efficacy while minimizing adverse effects. The strength of finerenone lies in its high selectivity for the mineralocorticoid receptor and its minimal cross-binding to other steroid hormone receptors under various physiological conditions. Additionally, its relatively balanced distribution between the kidneys and heart distinguishes finerenone from steroidal MRAs (44, 45). These pharmacologic properties contribute to the lower incidence of hyperkalemia and other adverse effects associated with finerenone compared with steroidal MRAs (46, 47). While there is no head-to-head randomized comparison between finerenone and steroidal MRAs in patients with CKD, an indirect *post hoc* analysis comparing 624 patients from the FIDELITY-TRH subgroup with 295 patients from the AMBER trial reported a markedly lower incidence of hyperkalemia with finerenone (48). The proportion of patients with serum potassium ≥ 5.5 mmol/L was 12% with finerenone and 3% with placebo, compared with 35% and 64% in those receiving spironolactone with and without patiromer, respectively (48). Treatment discontinuation due to hyperkalemia occurred in only 0.3% of patients on finerenone, versus 7 and 23% for spironolactone with and without patiromer, respectively (48). These findings suggest that although finerenone may still contribute to hyperkalemia in susceptible patients, its risk appears substantially lower than that associated with steroidal MRAs and should be weighed accordingly in clinical decision-making.

Finerenone is a dihydropyridine-based MRA with a distinct non-steroidal bulky structure that confers high selectivity for the MR without exhibiting activity at L-type calcium channels. This characteristic contributes to its hemodynamic stability by preventing disproportionate afferent arteriolar vasodilation. This was reflected in the FIDELIO-DKD and FIGARO-DKD trials, where finerenone produced a modest reduction in systolic blood pressure, with an average reduction of 2–3 mmHg compared with placebo (49, 50). Nevertheless, in a dedicated *post hoc* analysis of 624 patients with resistant hypertension and chronic kidney disease from the FIDELITY pooled analysis, finerenone was associated with a greater reduction in systolic blood pressure compared with placebo, with a mean difference of −5.74 mmHg (95% CI, −7.99 to −3.49; *P* < 0.0001) (48). By comparison, in the AMBER trial of 295 patients, spironolactone demonstrated a greater reduction in systolic blood pressure, with a mean decrease of −11.7 mmHg (95% CI, −14.1 to −9.3) when combined with patiromer and −10.8 mmHg (95% CI, −13.2 to −8.3) when administered without patiromer (48). Given that a substantial proportion of patients with chronic kidney disease have inadequately controlled blood pressure despite multiple antihypertensive therapies, the modest antihypertensive effect of finerenone should be taken into consideration, particularly when blood pressure control is a primary therapeutic objective.

**FIGURE 1:**
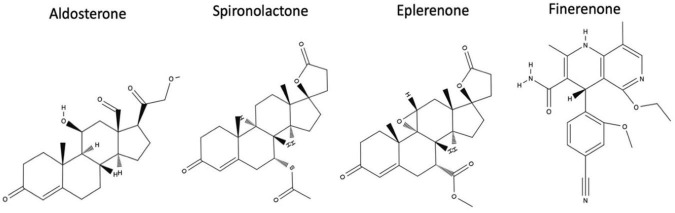
Chemical structures of aldosterone, spironolactone, eplerenone, and finerenone (54).

Its non-steroidal structure enables a more precise fit within the MR binding domain, enhancing its affinity and selectivity compared to glucocorticoid, androgen, and progesterone receptors. The bulky structure of finerenone enables the binding to the MR in a way that occupies the entire receptor, thereby preventing activation and blocking the MR from adopting an agonist conformation (Table 2). Additionally, the finerenone structure confers higher selectivity for the MR and minimizes off-target effects on androgen, progesterone, estrogen, and glucocorticoid receptors, which results in a lower incidence of adverse effects such as gynecomastia observed with steroidal MRAs like spironolactone (51). In contrast, spironolactone is a steroid antagonist characterized by a flat, card-like structure, high potency, and low specificity for the MR, while eplerenone is a steroid antagonist with reduced potency and moderate specificity for the MR (52). Finerenone exerts its therapeutic effects by competitively inhibiting MR in both epithelial tissues, notably the distal convoluted tubule 2 (DCT2), and non-epithelial tissues, thereby modulating sodium and water balance while attenuating pro-inflammatory and pro-fibrotic signaling (53). The chemical structures of finerenone and steroidal drugs are illustrated in Figure 1.

**TABLE 2:** Pharmacological properties of mineralocorticoid receptor antagonists (5, 44, 45, 55, 6).

Properties	Products		
	**Spironolactone**	**Eplerenone**	**Finerenone**
Pharmacological class, chemical structure	Steroidal, flat	Steroidal, flat	Non-steroidal, bulky
Potency to MR receptor	High	Low	High
Selectivity to MR receptor	Low	Medium	High
IC_50_ for MR receptor	24	990	18
IC_50_ for glucocorticoid receptor	2400	22,000	10,000 and up
IC_50_ for androgen receptor	77	21,200	10,000 and up
IC_50_ for progesterone receptor	740	31,200	10,000 and up
Half-life	>20 h	4–6 h	2–3 h
Tissue distribution	Kidney > > > Heart	Kidney > Heart	Kidney = Heart
Active metabolites	Multiple, active metabolites	Multiple, non-active	Non-active
Steroid-related side effects (e.g., gynecomastia)	Medium	Low	Low
Blood pressure effects	High	Medium	Minimal
FDA-Approved indications	Hypertension, heart failure, edema, primary hyperaldosteronism	Hypertension, heart failure post myocardial infarction	To improve kidney and CV outcomes in T2DM with CKD
Serum potassium level consideration (Expressed as mEq/L)	• Potassium > 5.0: consider reducing the dose to 25 mg every other day or discontinuing the medication, depending on the severity of the hyperkalemia.	• Potassium > 5.5: discontinue	Do not initiate if potassium > 5.0 for 10 mg daily dose: • Potassium ≤ 4.8: increase to 20 mg daily • Potassium > 4.8 to 5.5: maintain dose • Potassium > 5.5: hold finerenone; restart when potassium ≤ 5.0 for 20 mg daily dose: • Potassium ≤ 4.8: maintain dose • Potassium > 4.8 to 5.5: maintain dose • Potassium > 5.5: hold finerenone; restart at 10 mg daily when potassium ≤ 5.0
Monitoring frequency for serum potassium	• Monitor potassium within first week of initiation or dose adjustment, and regularly thereafter	• Monitor potassium within the first week of initiation and periodically thereafter. Monitor more frequent if at risk of hyperkalemia	• Measure potassium 4 weeks after initiation, then periodically. • More frequent monitoring may be needed if at risk of hyperkalemia
eGFR Consideration (mL/min/1.73 m^2^).	• eGFR 30–50: start cautiously at 25 mg every other day with close potassium and renal monitoring • eGFR < 30: not recommended	• eGFR 30–50: start cautiously at 25 mg every other day with close potassium and renal monitoring • eGFR < 30: not recommended	• eGFR ≥ 60: start with 20 mg once daily • eGFR 25–59: start with 10 mg once daily • eGFR < 25: not recommended

CKD, chronic kidney disease; CV, cardiovascular; eGFR, estimated glomerular filtration rate; MR, mineralocorticoid receptor; T2DM, type 2 diabetes mellitus.

## 4 Finerenone in diabetic kidney disease (DKD)

**FIGURE 2:**
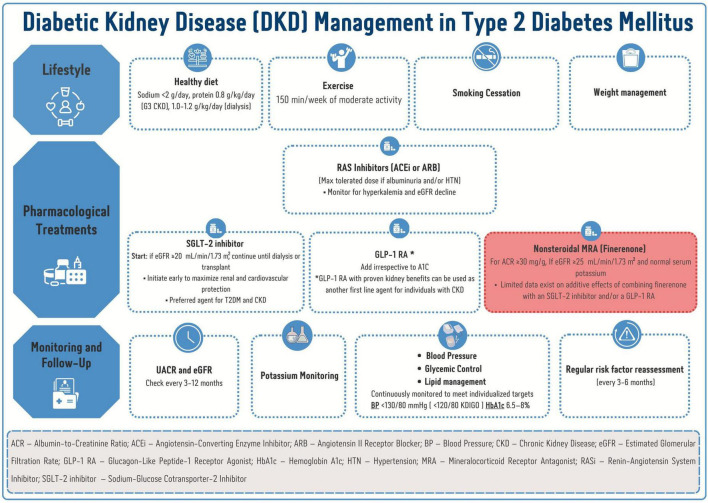
The place of finerenone therapy in the management landscape of diabetic kidney disease in patients with type 2 diabetes mellitus.

Finerenone introduces a new dimension in the management of DKD by selectively targeting MR–mediated pathways. Emerging clinical evidence demonstrates a novel therapeutic approach with unique pharmacologic properties to address the persistent kidney and cardiovascular risks that remain despite optimization of currently available therapies. Finerenone was approved by the United States FDA in 2021 to reduce kidney and cardiovascular risks in patients with T2DM and CKD (57). The ADA guideline recommends considering finerenone as an additional therapy for patients with T2DM and CKD who have an ACR of ≥30 mg/g and normal potassium levels of ≤4.8 mmol/L (12). The KDIGO guideline recommends using finerenone for patients with T2DM, an eGFR ≥ 25 ml/min/1.73 m^2^, normal serum potassium concentration, and albuminuria (>30 mg/g) despite a maximum tolerated dose of RAS inhibitor (58). Figure 2 shows the recommended place of therapy for finerenone within the therapeutic landscape of DKD in patients with T2DM.

The role of finerenone in DKD is supported by robust evidence from landmark clinical trials. The FIDELIO-DKD trial was the first study specifically designed to evaluate the long-term effects of finerenone on kidney and cardiovascular outcomes (49). This randomized, double-blind, placebo-controlled, multicenter trial included 5,734 adult participants diagnosed with T2DM and CKD, with a median follow-up duration of 2.6 years. The incidence of the primary composite outcome, which included kidney failure, a sustained decline of at least 40% in eGFR from baseline, or death from kidney causes, was significantly lower in the finerenone group compared to the placebo group. The primary outcome occurred in 504 patients (17.8%) in the finerenone group versus 600 patients (21.1%) in the placebo group, corresponding to a (HR 0.82; 95% CI: 0.73 to 0.93). Additionally, the risk of a sustained of at least 40% decline in eGFR was reduced in the finerenone group (HR 0.81; 95% CI: 0.72 to 0.92). For secondary outcomes, finerenone led to a 31% reduction in UACR compared to placebo at 4 months, an effect sustained throughout the study. While hyperkalemia occurred more frequently in the finerenone group (18.3% vs. 9.0% with placebo), treatment discontinuation due to hyperkalemia was relatively low, reported in 2.3% of patients receiving finerenone compared to 0.9% in the placebo group.

Complementing the findings of the FIDELIO-DKD trial, the FIGARO-DKD study further evaluated the efficacy and safety of finerenone, with a primary focus on cardiovascular outcomes in patients with CKD and T2DM (50). This multicenter, randomized, placebo-controlled trial enrolled 7,437 patients who were followed over a median of 3.4 years. The primary composite outcome—comprising death from cardiovascular causes, non-fatal myocardial infarction, non-fatal stroke, or hospitalization for heart failure—occurred in 12.4% of patients in the finerenone group and 14.2% in the placebo group (HR: 0.87; 95% CI: 0.76 to 0.98; *P* = 0.03). This benefit was primarily driven by a 29% reduction in hospitalization for heart failure (HR: 0.71; 95% CI: 0.56 to 0.90). While the study’s main focus was cardiovascular risk reduction, the secondary kidney composite outcome—defined as kidney failure, a sustained of at least ≥ 40% decline in eGFR, or death from kidney causes—occurred in 9.5% of patients receiving finerenone compared to 10.8% in the placebo group (HR: 0.87; 95% CI: 0.76 to 1.01). Although this difference did not reach statistical significance, it suggested a potential kidney benefit. This lack of significance may, in part, be attributed to the kidney composite outcome being assessed as a secondary endpoint rather than a primary focus of the trial, which may have influenced the study’s power to detect differences in kidney events. Adverse event rates were similar between groups, though hyperkalemia-related treatment discontinuation was more frequent in the finerenone group (1.2% vs. 0.4% with placebo).

Expanding on the foundational insights from the FIDELIO-DKD and FIGARO-DKD trials, the FIDELITY pooled analysis offered a comprehensive assessment of finerenone’s efficacy and safety across a broader population of patients with CKD and T2DM. This prespecified, patient-level meta-analysis integrated data from 13,026 participants enrolled from both FIDELIO-DKD and FIGARO-DKD trials (59). Over a median follow-up of 3.0 years, finerenone significantly reduced the risk of the composite cardiovascular outcome—including cardiovascular death, non-fatal myocardial infarction, non-fatal stroke, or hospitalization for heart failure—by 14% compared to placebo (HR: 0.86; 95% CI: 0.78 to 0.95; *P* = 0.0018). Additionally, the composite kidney outcome—defined as kidney failure, a sustained ≥ 57% decline in eGFR, or kidney death—was reduced by 23% in the finerenone group (HR: 0.77; 95% CI: 0.67 to 0.88; *P* = 0.0002). The analysis also highlighted a 30% reduction in the risk of a sustained ≥ 57% eGFR decline and a 20% reduction in ESKD events with finerenone compared to placebo. Importantly, while hyperkalemia was more frequent in the finerenone group (14.0% vs. 6.9% with placebo), treatment discontinuation due to hyperkalemia remained relatively low (1.7% vs. 0.6%). By synthesizing data from two landmark trials, FIDELITY demonstrates the dual cardiovascular and kidney benefits of finerenone and reinforces its role in comprehensive DKD management.

The FINEARTS-HF study explored the impact of finerenone on kidney outcomes in patients with heart failure with mildly reduced or preserved ejection fraction (HFmrEF/HFpEF) (60). This randomized, double-blind, placebo-controlled trial included 6,001 participants, with a mean baseline eGFR of 62 mL/min/1.73 m^2^, nearly half of whom had an eGFR below 60 mL/min/1.73 m^2^. Over a median follow-up of 2.6 years, the incidence of the composite kidney outcome—defined as a sustained ≥ 50% decline in eGFR, sustained eGFR decline to < 15 mL/min/1.73 m^2^, or kidney failure—was low and did not differ significantly between finerenone and placebo groups (2.5% vs. 1.8%, HR: 1.33; 95% CI: 0.94 to 1.89). Similarly, the more stringent kidney outcome of a sustained ≥ 57% decline in eGFR or kidney failure showed no significant difference (1.4% vs. 1.0%, HR: 1.28; 95% CI: 0.80 to 2.05). While finerenone led to an initial acute decline in eGFR during the first 3 months, the chronic eGFR slope thereafter was comparable to placebo, suggesting no long-term detrimental impact on kidney function. Notably, finerenone significantly reduced albuminuria, achieving a 30% reduction in UACR at 6 months compared to placebo (least-squares mean ratio 0.70; 95% CI: 0.67 to 0.73; *P* < 0.001). Additionally, finerenone lowered the risk of new-onset microalbuminuria and macroalbuminuria by 24% (HR: 0.76; 95% CI: 0.68 to 0.83) and 38% (HR: 0.62; 95% CI: 0.53 to 0.73), respectively. Although hyperkalemia was more frequent in the finerenone group, serious events leading to hospitalization or treatment discontinuation were rare. The FINEARTS-HF trial highlighted that although finerenone did not significantly affect definitive kidney outcomes in patients with HFmrEF or HFpEF, it led to meaningful reductions in albuminuria.

An important consideration in DKD management is the impact of combining SGLT-2 inhibitors, GLP-1 receptor agonists, or both with finerenone on background RAS therapies. Table 3 provides a summary of key clinical trials that involved finerenone. A dedicated trial investigating the combination of an SGLT-2 inhibitor with finerenone in patients with DKD is currently underway (CONFIDENCE; NCT05254002), which aims to provide valuable data for clinical practice. In the absence of direct clinical evidence, a simulated model-based study grounded in real trial data offers insights into the potential incremental benefits of combining these therapies. The study by Neuen et al. (61) presents a comprehensive modeling analysis estimating the long-term cardiovascular, kidney, and mortality benefits of combination therapy with SGLT-2 inhibitors, GLP-1 receptor agonists, and finerenone in patients with T2DM and albuminuria. Using actuarial modeling techniques, the study synthesized data from major randomized controlled trials to project potential lifetime outcomes compared to conventional care. The model focused on patients with T2DM and albuminuria to evaluate the effects of combination therapy on MACE, CKD progression, heart failure hospitalizations, and mortality. The findings suggest that triple therapy could substantially reduce adverse outcomes. Compared with conventional care, combination therapy was associated with a 35% reduction in the risk of MACE (HR 0.65; 95% CI 0.55 to 0.76) and a 58% reduction in CKD progression (HR 0.42; 95% CI 0.31 to 0.56). Cardiovascular mortality declined by 36% (HR 0.64; 95% CI 0.51 to 0.80), and all-cause mortality decreased by 33% (HR 0.67; 95% CI 0.55 to 0.80). These reductions translated into an estimated 3.2 additional years free from MACE and 5.5 years free from CKD progression in a 50-year-old patient cohort. The analysis also compared dual and triple therapy strategies, demonstrating incremental benefits with each additional agent. While the study’s projections are grounded in modeling rather than prospective trial data, it offers valuable insights into the potential of triple therapy with SGLT-2 inhibitors, GLP-1 receptor agonists, and finerenone, in addition to RAS blockade, to extend event-free survival and reduce mortality. Nonetheless, dedicated clinical trials are essential to validate these findings and inform evidence-based practice.

**TABLE 3:** Key clinical trials summary of finerenone.

Year/title	Sample size	Population	Inclusion criteria	Efficacy outcomes	Safety outcomes
2024 FOUNTAIN (real world data)	1,029	T2D with CKD	- Age ≥ 18 years - Two eGFR values between 15–60 mL/min/1.73 m^2^ - Two UACR measurements of ≥ 30 mg/g - Use of RAS blockers	- Due to short follow-up period, limit the assessment of efficacy outcomes.	- Hyperkalemia rate: 2.16–2.70 per 100 patients. - No hospitalizations due to hyperkalemia. Confidence interval, and P value were not provided in this observational analysis
2024 FINEARTS-HF	6,001	Heart failure (HFmrEF OR HFpEF)	- Age ≥ 40 years - Symptomatic heart failure - LVEF ≥ 40% - Elevated natriuretic peptides - Structural heart disease evidence	- During the first 3 months, finerenone led to an acute decline in eGFR of –2.9 mL/min/1.73 m^2^ (95% CI: –3.4 to – 2.4 mL/min/1.73 m^2)^ but did not alter chronic (from 3 months) eGFR slope (+ 0.2 mL/min/1.73 m^2^ per year; 95% CI: –0.1 to 0.4 mL/min/1.73 m^2^ per year), vs placebo. - Finerenone lowered risk of microalbuminuria by 24% (HR 0.76; 95% CI: 0.68–0.83) and macroalbuminuria by 38% (HR 0.62; 95% CI: 0.53–0.73).	- Hyperkalemia events: 9.7% in finerenone vs. 4.2% in placebo (HR 2.39; 95% CI: 1.97–2.91; *P* < 0.001). - Hospitalization due to hyperkalemia: 0.5% in finerenone group (HR 2.67; 95% CI: 1.07–6.63; *P* = 0.034).
2022 FIDELITY pooled analysis	13,026	Pooled data from FIDELIO-DKD and FIGARO-DKD	Same as in FIDELIO-DKD and FIGARO-DKD	- Finerenone reduced the risk of major kidney events (kidney failure, ≥ 57% eGFR decline, or renal death) by 23% (HR 0.77; 95% CI: 0.67–0.88; *P* = 0.0002). - Finerenone reduced ≥ 57% eGFR decline risk by 30% (HR 0.70; 95% CI: 0.60–0.83; *P* < 0.0001). - Finerenone reduced end-stage kidney disease risk by 20% (HR 0.80; 95% CI: 0.64–0.99; *P* = 0.040).	- Hyperkalemia events: 14.0% in finerenone vs. 6.9% in placebo (HR 2.17; 95% CI: 1.94–2.42; *P* < 0.001). - Discontinuation due to hyperkalemia: 1.7% in finerenone vs. 0.6% in placebo (HR 2.91; 95% CI: 2.01–4.21; *P* < 0.001). - Hospitalization or mortality rates were similar between groups (HR 0.96; 95% CI: 0.91–1.01; *P* = 0.087).
2021 FIGARO-DKD	7,437	CKD and T2D	- Age ≥ 18 years - CKD stages 2–4 (eGFR 25–90 mL/min/1.73 m^2^) with moderately elevated albuminuria - CKD stages 1–2 (eGFR > 60 mL/min/1.73 m^2^) with severely elevated albuminuria - On RAS inhibitor treatment - Serum potassium ≤ 4.8 mmol/L	- Finerenone reduced ESRD risk by 36% (HR 0.64; 95% CI: 0.41–0.995; *P* = 0.046). - Finerenone reduced UACR by 32% in 4 months (least-squares mean ratio 0.68; 95% CI: 0.65–0.70; *P* < 0.001).	- Discontinuation due to hyperkalemia: 1.2% in finerenone vs. 0.4% in placebo (HR 3.16; 95% CI: 1.68–5.94; *P* < 0.001).
2020 FIDELIO-DKD	5,734	CKD and T2D	- Age ≥ 18 years - Moderate albuminuria, with eGFR ≥ 25– < 60 mL/min/1.73 m^2^ and diabetic retinopathy - Severe albuminuria, with eGFR ≥ 25– < 75 mL/min/1.73 m^2^ - On RAS inhibitor treatment - Serum potassium ≤ 4.8 mmol/L	- Finerenone reduced the risk of major kidney events (kidney failure, eGFR decline, or renal death) by 18% (HR 0.82; 95% CI: 0.73–0.93; *P* = 0.001). - Finerenone was effective in slowing the progression of kidney disease, reducing the risk of significant kidney function decline (≥ 57% drop in eGFR) by 24% (HR 0.76; 95% CI: 0.65–0.90; *P* < 0.001). - Finerenone reduced UACR by 31% (least-squares mean ratio 0.69; 95% CI: 0.66–0.71; *P* < 0.001).	- Hyperkalemia events: 18.3% in finerenone vs. 9.0% in placebo (HR 2.18; 95% CI: 1.94–2.45; *P* < 0.001). - Discontinuation due to hyperkalemia: 2.3% in finerenone vs. 0.9% in placebo (HR 2.58; 95% CI: 1.65–4.04; *P* < 0.001). - Acute kidney injury was similar between groups (HR 0.96; 95% CI: 0.74–1.25; *P* = 0.76).

Several barriers limit the effective use of finerenone in managing DKD. Potential adverse effects, drug interactions, and specific eligibility parameters, such as albuminuria levels, eGFR thresholds, and serum potassium requirements may further limit its clinical use (62, 63). Clinical inertia can slow finerenone integration into treatment plans due to a lack of familiarity with its unique pharmacokinetics and pharmacodynamic properties as compared to the steroidal MRAs. A similar pattern of underuse has been observed with other newly approved therapies for DKD, such as SGLT-2 inhibitors. A cross-sectional analysis of approximately 1.2 million patients from the Veterans Health Administration Database revealed that only 12% of these patients were prescribed an SGLT-2 inhibitor (64). The safety profile of finerenone remained consistent across the studies analyzed. Hyperkalemia is a critical parameter for monitoring in patients receiving finerenone, particularly those with DKD. In landmark trials such as FIDELITY, the protocol for serum potassium monitoring included a baseline assessment before initiation, followed by re-evaluation 4 weeks after starting treatment, an interval less frequent than the typical initiation protocol for steroidal MRAs. The monitoring approach of hyperkalemia suggests a relatively favorable safety profile concerning hyperkalemia compared to steroidal MRAs. Overall, the safety profile of finerenone demonstrates a manageable incidence of hyperkalemia in patients with CKD and cardiovascular disease. However, it is essential to recognize the risk of hyperkalemia with finerenone, and this possibility should not be underestimated, especially if finerenone is combined with conventional therapies. Regular monitoring remains critical, particularly for patients at high risk.

## 5 Conclusion

Finerenone represents a novel therapeutic approach in the management of DKD by targeting distinct pathophysiological pathways not addressed by existing therapies. Landmark trials, including FIDELIO-DKD, FIGARO-DKD, and the pooled FIDELITY analysis, have established the efficacy of finerenone in reducing kidney disease progression and cardiovascular events in patients with T2DM and CKD. While current evidence demonstrates a manageable safety profile with finerenone, hyperkalemia remains a clinically relevant concern, particularly in patients at higher risk. Important questions remain regarding the long-term real-world effectiveness of finerenone and its potential synergistic effects when combined with other established therapies. Strategies to overcome barriers to access and uptake—such as clinician education, patient engagement, and cost considerations—will be critical in translating trial findings into widespread clinical practice.
